# Gene Annotation Easy Viewer (GAEV): Integrating KEGG’s Gene Function Annotations and Associated Molecular Pathways

**DOI:** 10.12688/f1000research.14012.3

**Published:** 2019-05-09

**Authors:** Trung Huynh, Sen Xu

**Affiliations:** 1 Department of Biology, University of Texas at Arlington, Arlington, Texas, 76019, USA

**Keywords:** molecular pathway, Daphnia, genome annotation, visualization, homologous genes

## Abstract

We developed a Gene Annotation Easy Viewer (GAEV) that integrates the gene annotation data from the KEGG (Kyoto Encyclopedia of Genes and Genomes) Automatic Annotation Server. GAEV generates an easy-to-read table that summarizes the query gene name, the KO (KEGG Orthology) number, name of gene orthologs, functional definition of the ortholog, and the functional pathways that query gene has been mapped to. Via links to KEGG pathway maps, users can directly examine the interaction between gene products involved in the same molecular pathway. We provide a usage example by annotating the newly published freshwater microcrustacean *Daphnia pulex* genome. This gene-centered view of gene function and pathways will greatly facilitate the genome annotation of non-model species and metagenomics data. GAEV runs on a Windows or Linux system equipped with Python 3 and provides easy accessibility to users with no prior Unix command line experience.

## Introduction

Describing the biological function of computationally annotated genes in non-model assemblies and the molecular pathways formed by these genes’ products is critical for identifying the genetic basis of the various unique biological attributes (e.g., physiology, life history, behavior) of the species in question. Computational search against DNA/protein databases, e.g., NCBI Blast ( Boratyn *et al.*, 2013), UniProt ( Bateman *et al.*, 2017), InterPro ( Finn *et al.*, 2017), based on homology and protein domain information using computational tools, such as Blast ( Camacho *et al.*, 2009), InterProScan ( Jones *et al.*, 2014), and Hmmer ( Mistry *et al.*, 2013), can make predictions for individual gene functions. In contrast, delineating the molecular pathways encoded by the entire suite of genes of a single species is a much more challenging task, especially for non-model species. To this extent, mapping genes to the molecular pathways derived from intensively studied model organisms provides an entry point for addressing this need.

For mapping genes into known molecular pathways, the Kyoto Encyclopedia of Genes and Genomes (KEGG) provide comprehensive web services ( Kanehisa *et al.*, 2017; Kanehisa & Goto, 2000; Kanehisa *et al.*, 2016a). KEGG is an integrated database for biological interpretation of genome sequences. The molecular function of genes is classified using ortholog groups, i.e., KEGG Orthology (KO). KEGG also contains KEGG pathways, BRITE hierarchies, and KEGG modules, all of which are networks of KO nodes. It is possible to annotate the molecular functions of a set of genes from complete/partial genome assembly or metagenomics dataset and their encoded molecular pathways using KEGG automatic annotation services that are provided through webservers BlastKOALA and GhostKOALA ( Kanehisa *et al.*, 2016b). For a non-model species, we can use KAAS (KEGG Automatic Annotation Server) web services to annotate the complete or random set of genes to describe their molecular function and map them into identified molecular pathways. The annotation results consist of KO numbers for each gene, genes mapped to KEGG pathway database, and genes mapped to BRITE. Nonetheless, the resulting complete set of pathways and BRITE hierarchy can only be viewed through the temporary URL provided by KEGG, which are only available for several days after the analyses are completed. Although these results are organized through either curated KEGG pathways or BRITE hierarchy, KAAS does not provide an integrative gene-centered view of gene function and pathways, i.e., the complete summary of gene function and all associated molecular pathways for each gene.

As can be envisioned, integrating the gene function annotation based on KEGG orthology and KEGG pathways can provide an efficient way to characterize both the predicted genes and associated pathways for a newly assembled genome or metagenomics dataset. Despite numerous computational packages for retrieving KEGG pathways using the API interface provided by KEGG database (e.g., Moutselos *et al.*, 2009; Wrzodek *et al.*, 2011), none of these packages to our best knowledge allows us to reconstruct the complete set of molecular pathways contained in a newly assembled genome. To provide a means to utilizing the highly informative resources at KEGG for annotating genomic sequences and molecular pathways for non-model species, we have developed a Gene Annotation Easy Viewer (GAEV) for integrating results of KEGG orthology annotation and KEGG pathways mapping using KEGG API tools in both Windows and Linux environment. GAEV is aimed to provide a gene-centered view of gene function and pathways, i.e., the complete summary of gene function and all possibly associated molecular pathways for each gene. This is distinct from other KEGG-related software such as MEGAN ( Huson *et al*., 2016) and MinPath ( Ye & Doak, 2009). MEGAN can achieve overall functional analysis of microbiome data with KEGG data ( Huson *et al*., 2016), whereas Minpath aims to provide a conservative and faithful estimation of the biological pathways for a query dataset ( Ye & Doak, 2009). GAEV is implemented in Python 3 and can be used as an independent package.

## Methods

**Figure 1.**
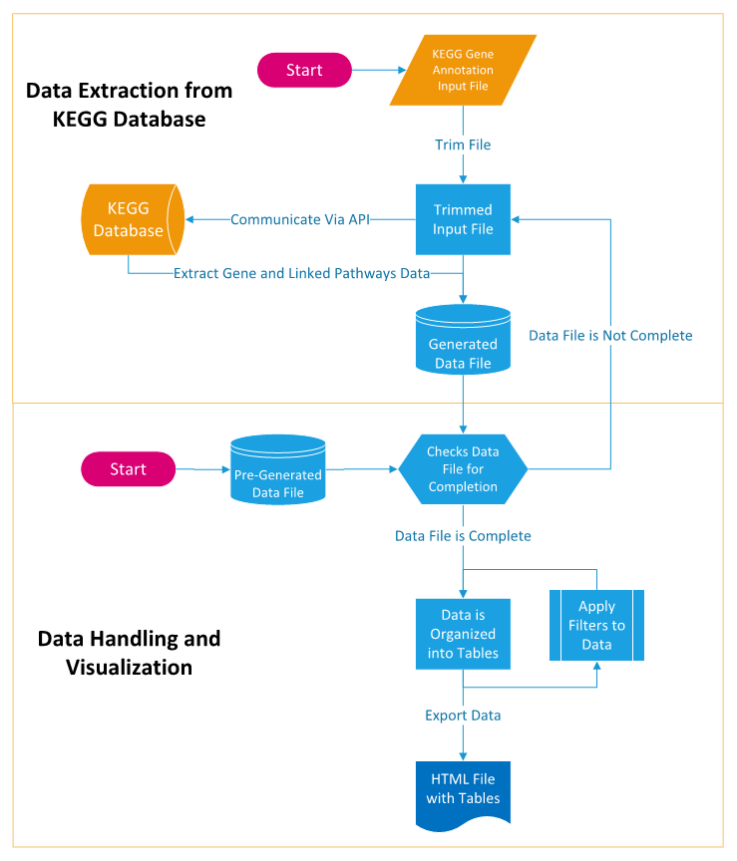
Workflow of Gene Annotation Easy Viewer (GAEV).

Assuming that the KEGG ortholog number is known for a single gene, the KO information can be retrieved from KEGG database by utilizing KEGG REST-style API. GAEV uses the ‘get’ operation of the KEGG API to extract data on the gene and linked pathways of every K number provided in the input file. The data extracted from KEGG database are stored in data files that can be loaded into GAEV to skip the data extraction step ( Figure 1).

Some genes will not have a KO number associated with them. Before data extraction, GAEV will trim the input file to remove all genes that do not have a KO number or have a KO number that cannot be searched in the KEGG database. Once data extraction from KEGG’s database is complete and the data file is generated, GAEV helps the user handle and visualize the data by exporting the data as a table in an HTML file. GAEV populates the table with the user defined gene ID provided in the input file and the associated K number provided in the input file, as well as the gene name, definition, and linked pathways that have been retrieved from the KEGG database. The linked pathway map URLs that highlight identified genes in the genome assembly are created using the following formula: http://www.kegg.jp/kegg-bin/show_pathway?map=[ **mapno]**&multi_query=%23bfffbf%0d%0a[ **k-num1**]+%23bfffbf%0d%0a **[k-num2]**+... %23bfffbf%0d%0a **[k-num_interest]+%**23[ **node_color**],%23 **[font_color]**.

In the above URL, **[mapno]** represents the pathway accession number. **[k-num{1,2,3…}]** represents the K number for each gene in the pathway that is present in the provided genome assembly, and **[k-num_interest]** represents the K number of the focal gene that will be highlighted with a unique color. **[node_color]** and **[font_color]** represent the desired color of the focal gene’s node and font on the pathway map, respectively. By default, the node color of the focal gene is dark red, whereas the node color of other genes in the same pathway that are present in the genome assembly is light green.

## Use cases

### Installation

The most up-to-date version of this software can be downloaded at https://github.com/UtaDaphniaLab/kegg_path_generator. This software requires Python 3 or newer to run. It is recommended that this software be used as a standalone program simply by double clicking on GAEV.py or by using the ‘python 3 GAEV.py’ command.

### Annotation

**Figure 2.**
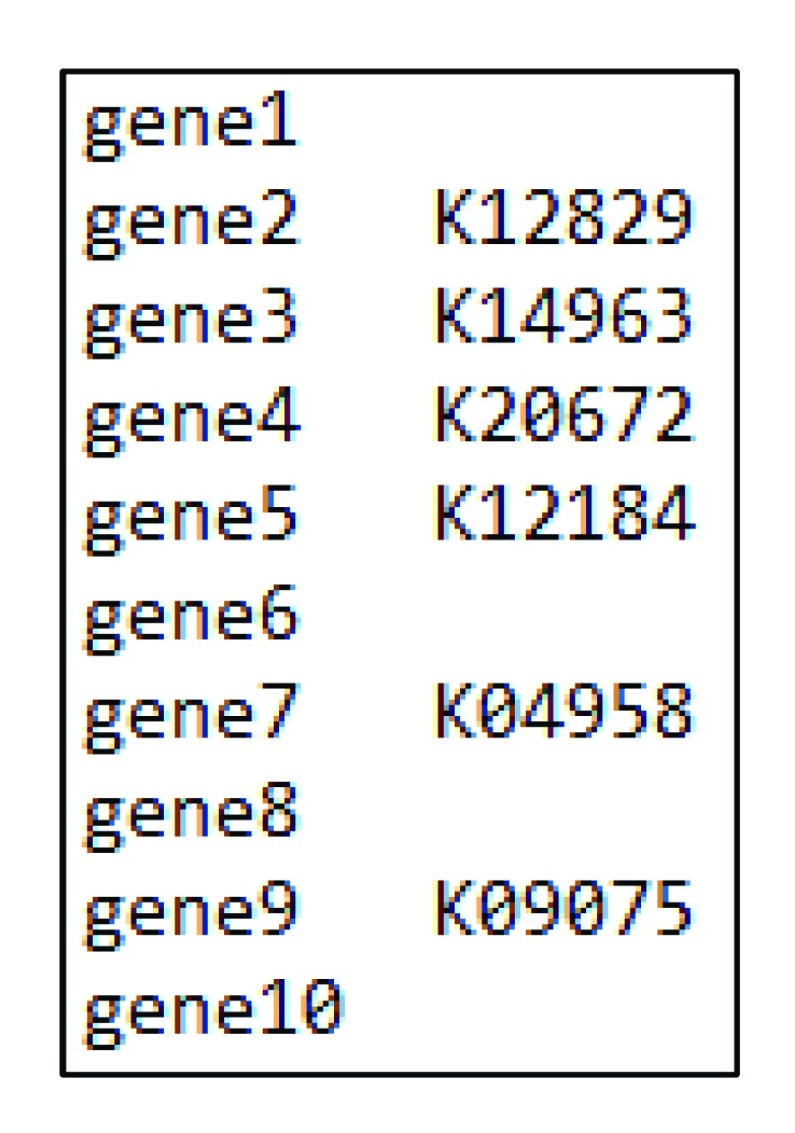
Example input file.

We analyze the newly published *Daphnia pulex* genome ( Ye *et al.*, 2017) to demonstrate the usage of our package. The required input file for our package contains two columns. The first column contains the gene names, whereas the second column represents the KO (KEGG orthology) numbers ( Figure 2, Supplementary File 1). The KO numbers for the entire set of genes can be obtained through KEGG Automatic Annotation Server. Briefly, users can provide the query protein sequences in a fasta file and use one of the provided search algorithms (e.g, Blast, GhostX, GhostZ) to assign KO numbers to each of the queried genes. The Daphnia protein fasta file can be found at https://figshare.com/articles/PA42_3_0_protein_new_txt/6653297. With a gff/gtf genome annotation file, users can also use tools such as gff2sequence ( Camiolo & Porceddu, 2013) to extract DNA/protein sequences from genomic assemblies, which can be used as query sequences. Furthermore, researchers working with non-model organisms could use protein sequences extracted from an assembled transcriptome as input data. At the end of this analysis, the user will receive via email a link to the result page, where the query result can be downloaded. The downloaded query result can be directly used as input file for our package even when some genes are not provided a KO number (which will be automatically excluded from further analysis).

With the obtained input file, the annotation analysis can be started by simply running GAEV.py and following the instructions of the menus. The first menu provides the option of using the obtained input file to extract data from KEGG or skipping the data extraction step by loading a pre-generated data file. Next, GAEV will prompt the user for the location of the input or data file. Both absolute and relative paths are accepted, but it is recommended that the GAEV.py file be placed in the same folder as the input or data file, so that the relative path can be easily used. After the data extraction from KEGG’s servers is completed, a data file will be created, which can be repeatedly used for making different pathway tables. The next several menus guide the user through the process of customizing the output table. The user has the options to apply filters so that GAEV only outputs a table using genes with a specific keyword in its definition or linked pathways.

### Output file

The output file is an html file that can be opened in any internet browser (for example see Supplementary File 2). The results are organized in three different sections. The first section is the Genes and Linked Pathways, where for each query gene the molecular function based on KO and relevant pathways are listed. For each gene, its associated pathway(s) contains a link to the corresponding pathway page on KEGG website, where this specific gene is colored in red and all the identified genes from the genome assembly are colored in green. The other two sections contain a list of the pathways sorted by the number of identified genes and by alphabetic order, respectively. These two sections provide a pathway-centered view of the functions of the annotated genome.

### Batch jobs

The batch functions located in the first menu can be used when there are several sets of genes in different input files that the user wants to annotate. The batch functions require a file with the relative/absolute paths of each input file on separate lines. Alternatively, entering ‘all’ will direct GAEV to run using every file with a txt extension as an input file if new data files need to be generated or every file with a dat extension if new tables need to be created from existing data files.

Filters can be applied to batch jobs and will apply to all sets of genes. An html output file like the one described above will be created for each set of genes in the same folder as its respective input and data file.

## Conclusions

**Figure 3.**
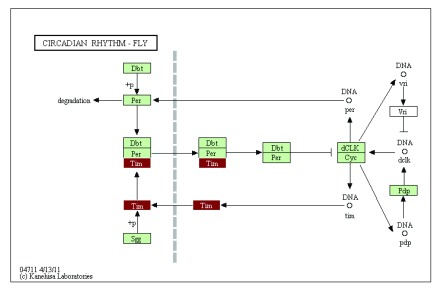
The circadian rhythm pathway in Daphnia pulex showing gene of interest (Tim) in red and other identified genes in green.

The integrative annotation approach implemented in our package GAEV draws upon resources available at KEGG and provides an efficient way to explore the molecular pathways embodied in a draft genome. The integration of the generated html file with KEGG web services provides an intuitive interface to explore specific molecular pathways, with all the identified KEGG homologs highlighted in the pathway map. This type of information is essential to initial exploration of non-model organisms’ genomes to understand the conservation of specific pathways compared to established model systems. For example, if we examine the circadian rhythm pathway in the *Daphnia* genome (by clicking on the link to the circadian rhythm pathway in the generated html file), we see strong conservation between *Daphnia* and *Drosophila*, with only 1 gene (i.e., *Vri*) in this pathway missing an identified homolog in the *Daphnia* assembly ( Figure 3). Further efforts can be dedicated to verifying the absence of *Vri* gene in *Daphnia* genome. The strong conservation of the circadian pathway can greatly aid future efforts in using the freshwater microcrustacean *Daphnia* to understand the internal clock of aquatic organisms in response to aquatic environments.

In principle, GAEV can be used for visualizing functions and pathways for gene sets of any scale, ranging from genome-wide data to subsets of genes in a genome. For example, we can use GAEV to visualize the pathways that differentially expressed genes are involved in. Often the large number of differentially expressed genes from RNA-seq experiments prevents clear cataloguing of these genes and molecular pathways. Analyzing the genes of interest using our package can provide a quick, integrative view of the genes and affected pathways.

In summary, with a user-friendly design (e.g., no requirement of UNIX command line experience) in mind, we have developed GAEV to provide a fast, easily accessible summary for KEGG gene annotation results. We expect that GAEV will find its use in many bioinformatic analyses, especially those involving non-model species. 

## Data and software availability

Software source code available from: https://github.com/UtaDaphniaLab/Gene_Annotation_Easy_Viewer

Archived source code as at time of publication: https://zenodo.org/record/2549592 ( Trung, 2019)

License: This software is licensed under the MIT license

## Supplementary files

Supplementary File 1. Example input file https://raw.githubusercontent.com/UtaDaphniaLab/Gene_Annotation_Easy_Viewer/master/gene_annotation_easy_viewer/example_input.txt

Supplementary File 2. Example output file https://raw.githubusercontent.com/UtaDaphniaLab/Gene_Annotation_Easy_Viewer/master/gene_annotation_easy_viewer/Example_Output.html
