# Supplementary material for: Gene Annotation Easy Viewer (GAEV): Integrating KEGG’s Gene Function Annotations and Associated Molecular Pathways
Source: F1000Res. 2019 May 9;7:416. Originally published 2018 Mar 29. [Version 3] doi: 10.12688/f1000research.14012.3 (PMC6343226; doi:10.12688/f1000research.14012.3)
Supplement: Peer review history

# Peer review: Reviewer response for version 3

**DOI:** 10.5256/f1000research.20342.r48234

**Published:** 2019-05-21

**Authors:** Tonia S. Schwartz

**Affiliations:** 1 Department of Biological Sciences, Auburn University, Auburn, AL, USA

The authors have addressed my comments. I have retested the script and it now works just as described. I think this manuscript provides a nice addition to the bioinformatic method amenable to non-model organisms.

I have read this submission. I believe that I have an appropriate level of expertise to confirm that it is of an acceptable scientific standard.

# Peer review: Reviewer response for version 3

**DOI:** 10.5256/f1000research.20342.r48232

**Published:** 2019-05-16

**Authors:** Simo V. Zhang

**Affiliations:** 1 Department of Computer Science, Indiana University Bloomington, Bloomington, IN, USA

I thank the authors for addressing all of my previous points. I have no additional comments and I recommend this manuscript to be indexed by the journal.

I have read this submission. I believe that I have an appropriate level of expertise to confirm that it is of an acceptable scientific standard.

# Peer review: Reviewer response for version 2

**DOI:** 10.5256/f1000research.16445.r42892

**Published:** 2019-01-21

**Authors:** Simo V. Zhang

**Affiliations:** 1 Department of Computer Science, Indiana University Bloomington, Bloomington, IN, USA

Huynh and Xu present a new approach, GAEV, to annotate gene models of a given non-model genome assembly. It maps genes to their associated pathway counterpart of model species. GAEV specializes on improving the conventional KEGG analyses and focuses on providing a gene centric view of gene function and pathways. Overall, I think GAEV can be a good addition to the current bioinformatics research community. I recommend indexing of this manuscript once the authors could address my following questions/suggestions. 

1. The first sentence of the manuscript confuses me about what problem the GAEV tries to solve. It provides a new and better way to annotate a given genome with pathways, but I don’t think it tries to help *de novo* assembly per se. I can see a possible role that GAEV plays in evaluating a non-model assembly by looking at how many conservative pathways and genes are completed (for example, using Drosophila’s circadian rhythm pathway to evaluate the newly assembled Daphnia genome). So it would be better if the authors could be clearer what problems GAEV tries to solve.
2. I have tested GAEV using the example data provided by the authors. It worked without any errors. However, I found two typos in the first two options of the menu popping up from typing “python GAEV.py”. Is it supposed to be “Create and generate a new data file and table from a new dataset of KO numbers”? Please fix it if it is the case.
3. GAEV provides an interactive interface to walk the users through the entire procedure. Such a design gives the users certain degree of flexibility. However, if some users need to try GAEV using different parameters or filters (which is usually true when one tries to annotate their genome/metagenome), such an interactive design can be overwhelming. I am wondering if the authors have plans to have an end-to-end automatic version of GAEV? By providing enough options from command line, I think GAEV can be both flexible and efficient. The likely problem with relative/absolute path to input files can also be naturally solved, and the requirement of putting the input files together with GAEV.py is not necessary as well. I am not asking the authors to have an implementation for this publication, but it would be nice to know the authors’ thoughts on this.
4. GAEV implements a Trimming method. However, I cannot find some descriptions of this step. Such information is very important to users to understand what has been done internally and how it has been done.
5. GAEV also supports “BATCH” mode. Could the authors provide some use cases and let the users know what they should expect in terms of the final HTML report?
6. One typo: “MEGAN can achieve overall functional analysisof microbiome …” should be “MEGAN can achieve overall functional analysis of microbiome …”.
7. In the URL formula, it seems that there are extra spaces after “www”. Please fix it if it is the case.

I have read this submission. I believe that I have an appropriate level of expertise to confirm that it is of an acceptable scientific standard, however I have significant reservations, as outlined above.

# Peer review: Reviewer response for version 2

**DOI:** 10.5256/f1000research.16445.r35571

**Published:** 2018-07-09

**Authors:** Fragiskos N Kolisis, Efthymios Ladoukakis

**Affiliations:** 1 Laboratory of Biotechnology, School of Chemical Engineering, National Technical University of Athens, Athens, Greece; 2 National Technical University of Athens, Athens, Greece

I have no further comments to make.

We have read this submission. We believe that we have an appropriate level of expertise to confirm that it is of an acceptable scientific standard.

# Peer review: Reviewer response for version 1

**DOI:** 10.5256/f1000research.15230.r32642

**Published:** 2018-05-02

**Authors:** Tonia S. Schwartz

**Affiliations:** 1 Department of Biological Sciences, Auburn University, Auburn, AL, USA

This is a review of the manuscript “Gene Annotation Easy Viewer (GAEV): Integrating KEGG’s Gene Function Annotations and Associated Molecular Pathways” by Huynh and Xu. The authors present a method via a python script that can take KEGG IDs and output three tables of useful annotation information in the context of the molecular pathways.

 The manuscript is well written and is easy to read. I envision that the tool developed will be useful, although see the comments below. With corrections I think this manuscript will be a good contribution to the resources available to researchers working in genomics/transcriptomics in non-model organisms.

 Major Comments

 Although I can start the python script running using python3, the example_input.txt file cannot be found when I enter the relative path or the absolute path. This is despite the input file being in the same folder as the script. My bioinformatics technician also encountered the same problem. Thus it seems there is an error in the script that would need to be corrected prior to acceptance of the manuscript.

 Minor Comments

 This tool is presented as accessible and user-friendly, and the authors use the example from Ye et al. 2017 of  “the query protein sequences in a fasta file”. But a protein sequence file is not provided with that paper (that I could find). Thus there an assumed knowledge gap that your reader needs to be able to use this tool as you describe. I suggest you provide additional information on how users can go from a genome with predicted genes (i.e. a .gtf or .gff3 file) to obtain those query sequences for input into the KEGG. 

 You may also want to mention that researchers working with non-model organisms could start with a de novo transcriptome or as an input file.

I have read this submission. I believe that I have an appropriate level of expertise to confirm that it is of an acceptable scientific standard, however I have significant reservations, as outlined above.

# Response

**Published:** 2018-05-23

**Authors:** Trung Huynh

**Affiliations:** University of Texas at Arlington, USA

Dear Dr. Schwartz,

 Thanks very much for your comments on our manuscript. Please see below for how we revised our manuscript to address your concerns.

*1. Although I can start the python script running using python3, the example_input.txt file cannot be found when I enter the relative path or the absolute path. This is despite the input file being in the same folder as the script. My bioinformatics technician also encountered the same problem. Thus it seems there is an error in the script that would need to be corrected prior to acceptance of the manuscript.*

**Response**: We have found the bug in our code that prevented the script from finding files on Linux and Mac OS properly. This bug has been fixed as of version 1.1.1.

*2. This tool is presented as accessible and user-friendly, and the authors use the example from Ye et al. 2017 of  “the query protein sequences in a fasta file”. But a protein sequence file is not provided with that paper (that I could find). Thus there an assumed knowledge gap that your reader needs to be able to use this tool as you describe. I suggest you provide additional information on how users can go from a genome with predicted genes (i.e. a .gtf or .gff3 file) to obtain those query sequences for input into the KEGG.*

**Response**: We provide a fasta file of Daphnia protein sequence through the github page for GAEV. Moreover, in the manuscript we recommend users to use tools such as gff2sequence to create query sequences using information from gtf/gff files. See 2 ^nd^ paragraph of Use Cases.

*3. You may also want to mention that researchers working with non-model organisms could start with a de novo transcriptome or as an input file.*

**Response**: Addressed accordingly. See 2 ^nd^ paragraph of Use Cases.

# Peer review: Reviewer response for version 1

**DOI:** 10.5256/f1000research.15230.r32636

**Published:** 2018-04-16

**Authors:** Fragiskos N Kolisis, Efthymios Ladoukakis

**Affiliations:** 1 Laboratory of Biotechnology, School of Chemical Engineering, National Technical University of Athens, Athens, Greece; 2 National Technical University of Athens, Athens, Greece

The authors of this article present a new tool, compiled as a single Python script, that aids in visualizing the gene annotation analysis results of the webserver-based KEGG services. The function of this tool is to generate an html-based view of the genes analyzed by KEGG tools and the appropriate pathways with which they are linked. The html view provides also an overview of the total existing pathways per associated genes, a task which can be useful for whole genome and metagenome annotation queries.

 The authors provide a thorough explanation about how the tool works and communicates with KEGG API by generating the appropriate links and exporting the generated data. The authors also provide some test datasets which can be used to generate the results mentioned in the manuscript.

 Nevertheless this reviewer considers this endeavour to have already been covered by other bioinformatic tools with which a comparison would be necessary to underline the importance of the new tool. For example tools like MEGAN can provide a thorough investigation of the existing KEGG pathways in a genome/metagenome (although by using an older and not commercial version of the KEGG database). Furthermore MinPath can be also used in combination with KEGG generated datasets in order to provide a similar pathway reconstruction analysis. Maybe the authors could elaborate a little bit more about what makes their tool more suitable than these already published tools.

 Moreover during the Conclusions section the authors do not seem to explain the methodology in order to examine the differences between Daphnia and Drosophila and how that (and similar analyses) can be achieved solely (or more intuitively) by exploiting this particular tool.

 In general this tool seems like a good addition to a bioinformatic pipeline for genomic or metagenomic analysis but this reviewer thinks that the author must emphasize more on the differences and/or improvements regarding similar tools.

We have read this submission. We believe that we have an appropriate level of expertise to confirm that it is of an acceptable scientific standard, however we have significant reservations, as outlined above.

# Response

**Published:** 2018-05-23

**Authors:** Trung Huynh

**Affiliations:** University of Texas at Arlington, USA

Dear Dr. Kolisis,

 Thanks very much for your comments on our manuscript. Please see below for how we revised our manuscript to address your concerns.

 1. *Nevertheless this reviewer considers this endeavour to have already been covered by other bioinformatic tools with which a comparison would be necessary to underline the importance of the new tool. For example tools like MEGAN can provide a thorough investigation of the existing KEGG pathways in a genome/metagenome (although by using an older and not commercial version of the KEGG database). Furthermore MinPath can be also used in combination with KEGG generated datasets in order to provide a similar pathway reconstruction analysis.*

**Response**: Our goal with our new tool is to provide a gene-centric view of molecular pathways, where each gene is accompanied by all the pathways where this gene is predicted to play a role. This is different from the purposes of MEGAN and Minpath. We emphasized this idea in the last paragraph of Introduction and drew comparison with MEGAN and Minpath.

 2 *. Moreover during the Conclusions section the authors do not seem to explain the methodology in order to examine the differences between Daphnia and Drosophila and how that (and similar analyses) can be achieved solely (or more intuitively) by exploiting this particular tool.*

**Response**: This example of *Daphnia* circadian pathway is to demonstrate using this tool for initial exploration of non-model organisms’ genomes to understand the conservation of specific pathways compared to established model systems (i.e., Drosophila). The Drosophila circadian pathway is provided through KEGG database. Users can directly examine their interested pathways from the results of GAEV (click on the link in the generated html file) and view the pathways and mapped genes on KEGG website. We provide a brief explanation of how to technically view the pathway on KEGG server in Discussion.
